# A pilot study of marking accuracy and mental workload as measures of OSCE examiner performance

**DOI:** 10.1186/s12909-016-0708-z

**Published:** 2016-07-25

**Authors:** Aidan Byrne, Tereza Soskova, Jayne Dawkins, Lee Coombes

**Affiliations:** 1 Abertawe Bro Morgannwg University Local Health Board, Swansea, UK; 2 Cardiff University, School of Medicine, Cardiff, UK; 3 Department of Anaesthesia, Morriston Hospital, Swansea, SA6 6NL UK

**Keywords:** Mental workload, OSCE, Examiner training, Examiner accuracy

## Abstract

**Background:**

The Objective Structured Clinical Examination (OSCE) is now a standard assessment format and while examiner training is seen as essential to assure quality, there appear to be no widely accepted measures of examiner performance.

**Methods:**

The objective of this study was to determine whether the routine training provided to examiners improved their accuracy and reduced their mental workload. Accuracy was defined as the difference between the rating of each examiner and that of an expert group expressed as the mean error per item. At the same time the mental workload of each examiner was measured using a previously validated secondary task methodology.

**Results:**

Training was not associated with an improvement in accuracy (*p* = 0.547) and that there was no detectable effect on mental workload. However, accuracy was improved after exposure to the same scenario (*p* < 0.001) and accuracy was greater when marking an excellent compared to a borderline performance.

**Conclusions:**

This study suggests that the method of training OSCE examiners studied is not effective in improving their performance, but that average item accuracy and mental workload appear to be valid methods of assessing examiner performance.

**Electronic supplementary material:**

The online version of this article (doi:10.1186/s12909-016-0708-z) contains supplementary material, which is available to authorized users.

## Background

The Objective Structured Clinical Examination (OSCE) is commonly used as the method of assessing clinical skills [1]. Despite the high stakes nature of these assessments, doubt has been raised as to whether the format does provide an entirely objective assessment of student performance [2–4].

While there are accepted measures use to quality assure an OSCE [5] such as Inter-Rater Reliability (IRR), this quantifies the level of agreement between examiners, but does not exclude the possibility that examiners are consistently failing to use the objective criteria and instead providing global, subjective ratings [6, 7]. Calculated parameters such as MDiff (mean difference), [8] MACD (Mean Absolute Checklist Difference) [8] and Agreement Rate (AR), [9] have been suggested as more detailed assessments of examiner performance, but all assume the use of a dichotomous checklist and average the performance of an examiner over multiple stations. Generalizability theory can also provide a method of quantifying error [8] but should only be used where large datasets are available. Further, these methods have the fundamental problem that they rely on post hoc calculation which can only identify poorly performing examiners after the assessment has been completed. We were unable to identify any criteria validated as a method of identifying prospective examiners as competent to take part in a high stakes assessment.

Although examiner inaccuracy appears to be an accepted problem, [10] there has been little investigation into why inaccuracies occur. The field of Human Factors, defined as “the characteristics of human beings that are applicable to the design of systems and devices” [11], provides a wealth of techniques to identify the causation of such problems and such an approach suggests that the cognitive requirements of marking an OSCE could exceed the capacity of examiners [12, 13]. That is, it suggests that the human mind is not capable of accurately observing the actions of a student, evaluating their performance against fixed criteria and then accurately recording the result in the time provided. If so, then examiners might to resort to making more subjective, global assessments. This concept has been supported by a pilot study which indicated that the mental workload of OSCE examiners was indeed excessive [12].

While mental workload is an abstract concept, it can be seen as the proportion of an individual’s ability to process information in use at any point in time, with excessive workload associated with poor performance/error. In contrast, expertise is characterised by the ability to perform complex tasks with low levels of mental workload. The measurement of mental workload is it is well established in many high risk industries [14] and increasingly in the medical setting [15]. The secondary task method involves providing operators with a very simple additional (secondary) task such as responding to a simple stimulus in addition to the primary task such as assessing students during an OSCE. If the participant is trained to complete the primary task (low mental workload), then they would be expected to complete the secondary task accurately. However, if participants were overloaded, performance of the secondary task would be expected to deteriorate. As the secondary task method only identifies periods of cognitive overload, any deterioration in secondary task performance is usually equated to cognitive overload and a risk of poor performance [16].

When used as a training outcome, it would be usual to train participants until their expertise increased to the point where they were able to complete both the primary and secondary tasks accurately to confirm that there was no evidence of cognitive overload [17].

The above suggests that if OSCE examiners are provided with a simple, secondary task at the same time as marking the performance of video recorded student performances, it would be expected that an untrained examiner would perform the secondary task poorly and record an inaccurate assessment. In contrast, an adequately trained examiner would be expected to perform the secondary task effectively and to mark accurately, so that the performance of examiners could be determined prior to participation in a high stakes assessment.

Our hypothesis was therefore that the routine training provided to OSCE examiners would be effective in changing their performance from that of a novice to that of an expert and that this would be evident by their marking accuracy and secondary task performance improving as their expertise increased and their mental workload decreased.

## Methods

After institutional ethical approval, prospective examiners in a single Graduate Entry Medical School were studied during their initial training session. The assessment instrument for which they were being trained was an OSCE, used as a summative assessment of the clinical skills of medical students, including practical, examination and communication skills. Each station focussed on a single skill with the performance of each student compared to a list of 20–25 items. The instrument offered examiners a variety of options for each item, with some items scored as 1/0 (completed/not done), some as 2/1/0 (completed/partially completed/not done) and some as 3/2/1/0 (completed/partially completed/attempted/not done) as described elsewhere [18]. Examiners were not provided with specific anchors/grade descriptors for performances in the mid-range, but were expected to make personal judgements. The scores for each item were added to produce a total score for each candidate/station. The passing score for each station was determined by the borderline regression method [19].

The examiner training programme had been in place for four years and lasted around two and a half hours with 4–6 participants in each training session. Each session started with an hour long slide based presentation explaining the OSCE process, marking, standard setting and student feedback delivered by an experienced examiner. After discussion, each subject completed four cycles of simulated marking practice using video recorded performances and the appropriate checklist. After each practice, participants compared marks with their peers, discussed the process and were provided with feedback from the same experienced examiner. Two videos were based on the same communication (history taking) scenario and two contained the same (cardiovascular) examination scenario. Each scenario had one video of an excellent performance and one of a borderline fail performance. Completion of the training was regarded by the institution as evidence of competence. As a pilot study had identified a marked effect of prior exposure to a case, the order of the cases were reversed for half the training sessions (Table 1), however, all participants viewed the same four videos.

**Table 1:** Summary of cases used. (Good/Borderline fail refers to the performance level of the student being assessed)

		Video presentation			
		1	2	3	4
Group 1 (*n* = 10)	Theme	Cardiovascular examination	Cardiovascular examination	History taking	History taking
	Standard	Excellent	Borderline fail	Excellent	Borderline fail
Group 2 (*n* = 10)	Theme	History taking	History taking	Cardiovascular examination	Cardiovascular examination
	Standard	Borderline fail	Excellent	Borderline fail	Excellent

Examiners were selected on an opportunistic basis as training sessions were run, with each examiner required to provide written consent prior to participation. All examiners were qualified medical practitioners in either community based or hospital practice and all were actively involved in student teaching. No participant declined to take part in the study.

Prior to the start of the study, three tutors who had designed the training were asked independently to rate the performance recorded in each video using the standard marking checklist. The ‘correct’ answer for each item in the checklist was taken as the agreed response by all three tutors, or, in the small number of items where there was a discrepancy, the majority response was taken as correct, a method previously described by De Champlain et al. [9].

Prior to starting the training a vibrotactile device was attached to upper arm of each participant which vibrated at random, at intervals of between 10 and 90 s. They were asked to tap the screen of the device as soon as possible to terminate the vibration as a secondary task. The time at which the stimulus was delivered and the response time for each participant were logged automatically by the device for later analysis.

At the end of the session, response time data and checklist scores were collected and entered into a single database (Microsoft ACCESS, Redmond, Washington).

The completed checklists from each participant were compared to the ‘correct’ responses with the difference between the two recorded as error, ignoring whether the error was positive or negative. The Average Error per Item was calculated as the total error per case divided by the number of items in the checklist, as a modification of the method described by De Champlain et al. [9].

Using the previously validated methodology for measuring mental workload, [20] any response to the vibration stimulus of more than the upper limit of normal (1350 ms) was regarded as evidence of cognitive overload. For each stimulus the delay was calculated as response in ms minus 1350 ms, with all normal responses taken as zero. The Average Delay in each case was calculated as the total delay divided by the number of stimuli.

The normality of distributions was assessed with a Shapiro-Wilk test with the significance of differences between groups tested by one way ANOVA using SPSS (IBM Corp. Version 20.0. Armonk, NY).

## Results

In all cases the examiners completed each of the four assessments and data from the assessments and response times were all available for analysis.

In terms of overall mark awarded by an examiner when compared to the correct mark, the median error was 4 (7.0 %) Marks, interquartile range 2–4 (3.5–7.0 %), range 0–15 (0–26 %), with 56.3 % of marks lower than the standard. There were no significant differences in the overall mark awarded between examiners, scenarios or number of attempts.

**Fig. 1:**
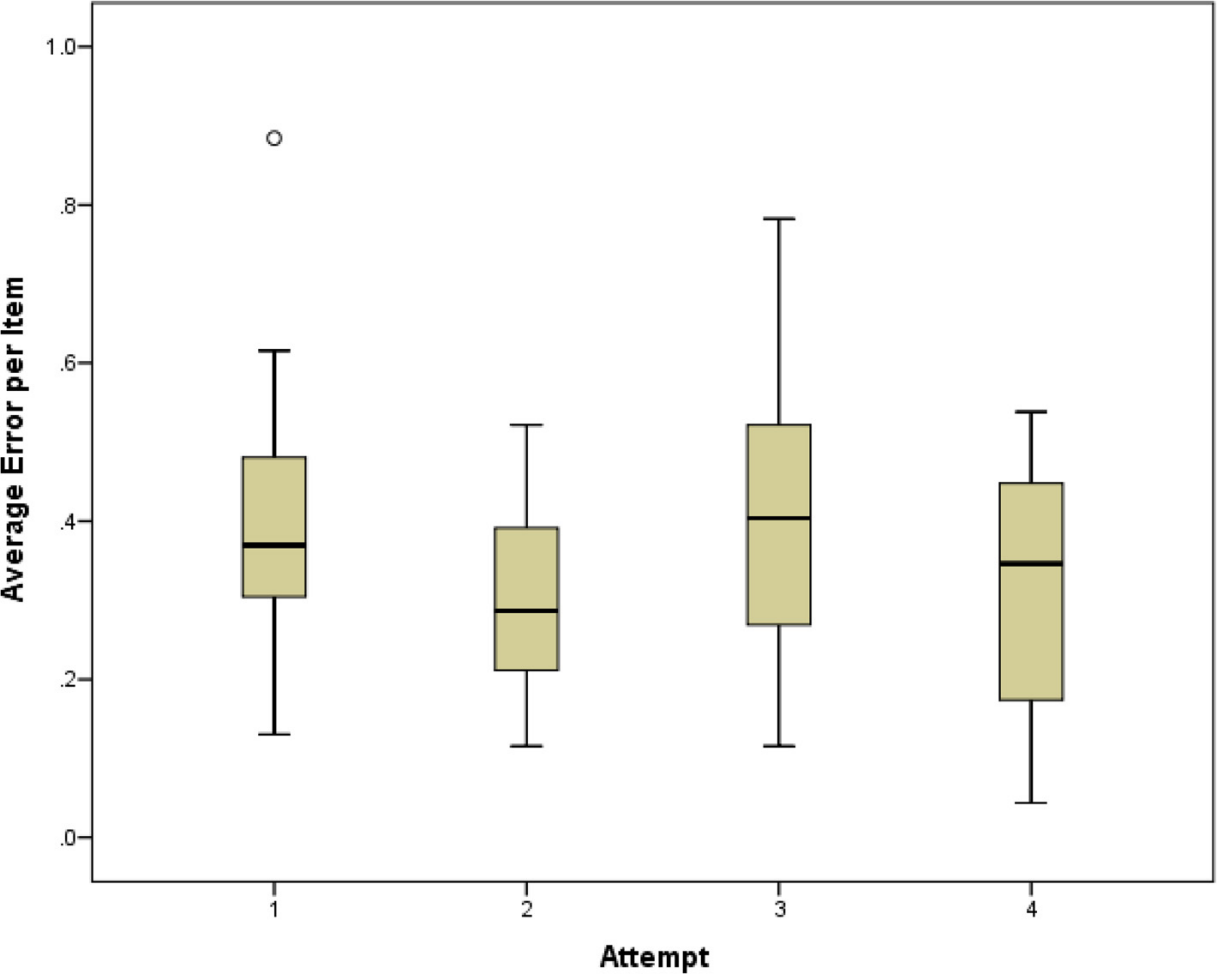
Mean Average Error per Item for all Participants across all four training scenarios. Differences between groups was not significant (*F* = 2.383, *P* = 0.076)

The distribution of Average Error per Item appeared to have a normal distribution confirmed by a Shapiro-Wilk analysis (S = 0.976, df = 80, *p* = 0.142). The Average Error per Item was 0.35 (range 4.34–0.88, SD 0.165, *n* = 80). One way Anova analysis including Average Error per Item as the dependant variable with number of attempts [1–4], type of scenario (History Taking/Cardiovascular Examination), student performance (Excellent/Borderline Fail) and prior exposure to scenario (Yes/No) as independent variables. The Average Error per Item did not change significantly from attempt 1 to 4, so that there was no evidence that training improved performance (Fig. 1). Although the type of scenario (History or Examination) had no significant effect on the Average Error per Item, it was significantly increased where the recorded performance was Borderline and where the examiner had prior experience of that scenario (Table 2).

**Table 2:** Summary data of mean error per Item

	Groups	Mean error per item	Standard deviation	F	P
Scenario	History taking	0.37	0.171	0.366	0.547
	Cardiovascular examination	0.34	0.160		
Student performance	Borderline fail	0.47	0.111	68.60	< 0.001
	Excellent	0.24	0.131		
Prior exposure	Yes	0.31	0.138	7.313	=0.008
	No	0.40	0.178		

**Fig. 2:**
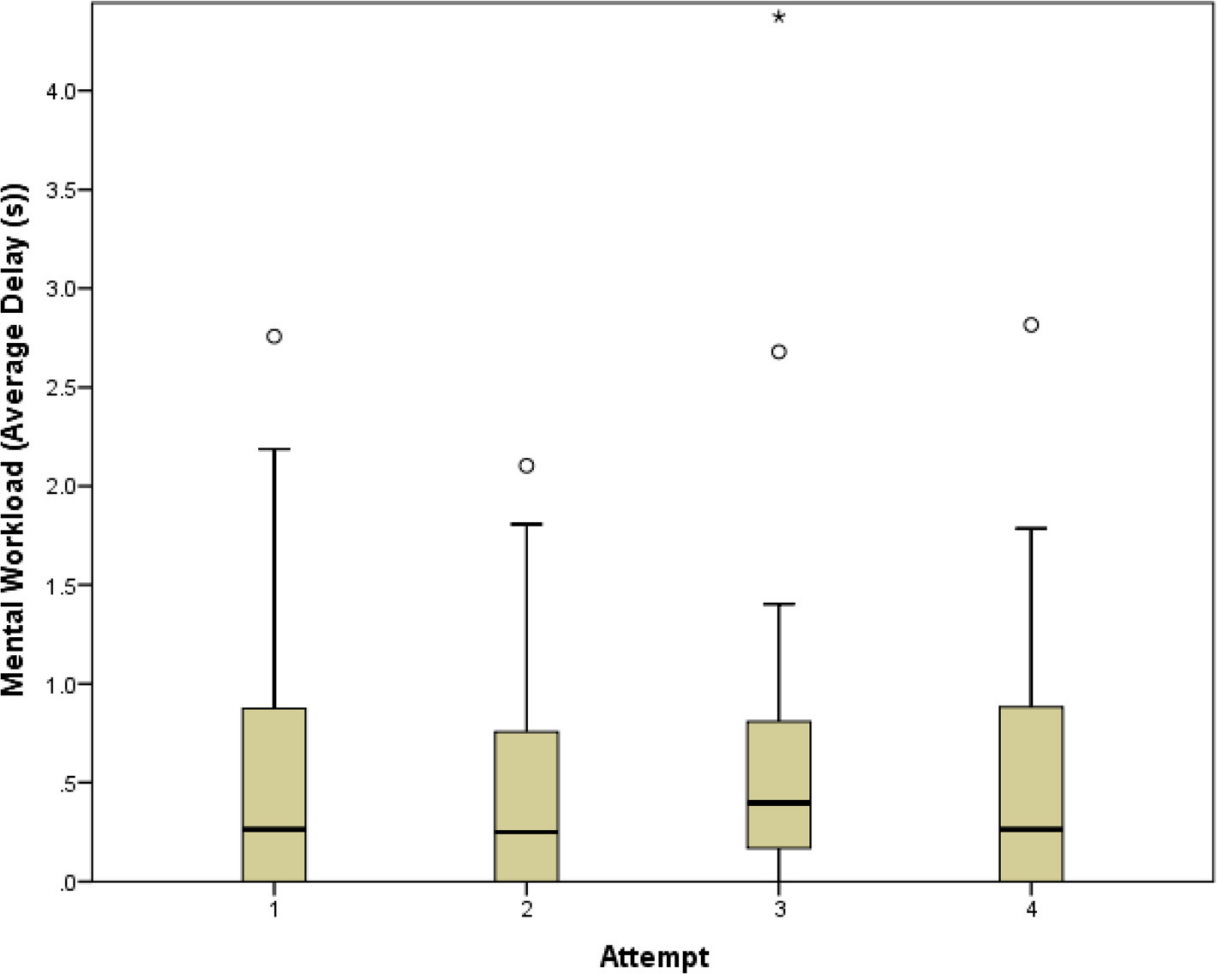
Mental workload for all participants

The mental workload as measured by Average Delay was consistent with periods of cognitive overload similar to the previous pilot study (Fig. 2) [12]. Although there appeared to be a small decrease to mental workload associated with the second and fourth cases, there were no statistically significant differences.

## Discussion

The principle finding of this study is that training OSCE examiners using the training described appeared to have no detectable effect on either their marking accuracy or their mental workload. This is surprising as the training was perceived by both staff and participants as highly effective. Given the error rates detected and that the checklists had 24/26 items with a total 57 marks available for both scenarios, the observed median error of 7 % and maximum error of 26 % would appear to be highly educationally significant. The observed level of mental workload would be consistent with cognitive overload and could explain some or all of the observed error [13].

While these results might suggest that either this form of training is entirely ineffective or that the role of an OSCE examiner is beyond the cognitive abilities of most humans, we would instead postulate that the process of OSCE marking should be regarded as a skill, which requires ‘sustained, deliberate practice’ [21] for the examiners to develop appropriate levels of expertise. If true, we would only expect trained and experienced examiners to provide truly objective assessments. This hypothesis would explain findings from other studies in that the performance of OSCE examiners would improve during a long examination process even if feedback was not provided, [22] and that an increase in the complexity of the checklist would be associated with a decrease in accuracy (82.75 % with 5 items to 76.19 % with 30 items) [23].

The increased examiner error where student performance was Borderline Fail may be explained by the need for borderline performances to be accurately compared to the required standard and an appropriate middle rating chosen, whereas Excellent/Clear Fail performance requires the less onerous task of selecting the highest/lowest available rating. However, if true, it would suggest that the OSCE format is least reliable where student performance is borderline.

The reduction in error after exposure to a specific scenario, but not an unrelated scenario suggests that there is an effective learning process at work, but that the learning is highly context specific [24]. This suggests that training examiners on the scenario they are to mark in a later summative assessment may be more effective than training on unrelated scenarios. However, a combination of factors, such as the checklist design, the clinical scenario, the performance level of the student, the expected performance level may also affect examiner performance. Overall, these data suggest that the incorporation of marking accuracy into the training process could both alert subjects to their own error and identify subjects whose performance might not be acceptable prior to their participation in a summative assessment. The use of mental workload as an outcome may also aid the redesign of the assessment process to improve the performance of examiners.

This study was limited as it included only small numbers of participants and to one form of training in a single Medical School. The results may not generalise to other institutions and differences in the OSCE format, in particular, the use of a dichotomous rating scale might be expected to require less cognitive workload. It is also possible that some aspect of the presentation itself was the important factor, for example, it might suggest that watching a video recording might require the acquisition of new skills in staff familiar with the observation of real clinical events. In addition, although these data suggest that training does not improve the performance of OSCE examiners in later, real assessments, this was not tested. While the performance of participants on subsequent scenarios did not improve, it may improve after later briefings or practice. The lack of change in measured mental workload may be a reflection of the small sample size and does not exclude the possibility that mental workload may change with later practice.

## Conclusions

This study supports the concept of OSCE examination as a high mental workload task and that a single training session does not reliably train examiners to the standard required to provide an accurate, objective assessment of student performance. However, the use of error per item and secondary task technique appear to provide a new methodology for the further investigation of the performance and training of OSCE examiners.

## Abbreviations

AR, Agreement Rate; IRR, inter rater reliability; MACD, Mean Absolute Checklist Difference; MDiff, mean difference; OSCE, Objective Structured Clinical Examination

## Additional file

Additional file 1: Response time data, OSCE Scores of subjects, Max available scores and Expert scores. Response Time Data – Unique Identifier, Subject Number, Date, Time, Response time in seconds, Phase (1 = listening, 2 = rating video), Video = number of video being rated. OSCE Scores – Unique identifier, Scenario (CVS = cardiovascular exam, Comm = Communication Skills, P = Borderline Pass, G = Good performance), Subject Number, Attempt (number 1–4), Item on Checklist, Score as recorded by subject. OSCE Max Scores available – Unique Idenifier, Subject (blank), Attempt (not relevant here), Item on Checklist, Score – Max score available. Expert Scores – Agreed scores for each item on checklist for each of the four videos used, Unique identifier, Scenario as previously, Subject (blank), Attempt (not relevant here), Item on Checklist, Agreed Score. (XLSX 158 kb)

## References

[1] Harden RM, Stevenson M, Downie WW, Wilson GM (1975). Assessment of clinical competence using objective structured examination. Br Med J.

[2] Lafleur A, Côté L, Leppink J (2015). Influences of OSCE design on students’ diagnostic reasoning. Med Educ.

[3] Yeates P, Moreau M, Eva K. Are Examiners' Judgments in OSCE-Style Assessments Influenced by Contrast Effects?Acad Med. 2015;90(7):975-80. 10.1097/ACM.0000000000000650 25629945

[4] Park WB, Kang SH, Lee Y-S, Myung SJ. Does Objective Structured Clinical Examinations Score Reflect the Clinical Reasoning Ability of Medical Students?Am J Med Sci. 2015;350(1):64-7. 10.1097/MAJ.0000000000000420 PMC4495861 25647834

[5] Pell G, Fuller R, Homer M, Roberts T (2010). How to measure the quality of the OSCE: a review of metrics – AMEE guide no. 49. Med Teach.

[6] Cunnington JPW, Neville AJ, Norman GR (1996). The risks of thoroughness: reliability and validity of global ratings and checklists in an OSCE. Adv Health Sci Educ.

[7] Daniels V, Bordage G, Gierl M, Yudkowsky R (2014). Effect of clinically discriminating, evidence-based checklist items on the reliability of scores from an Internal Medicine residency OSCE. Adv Health Sci Educ.

[8] Boulet J, McKinley D, Whelan G, Hambleton R (2003). Quality assurance methods for performance-based assessments. Adv Health Sci Educ.

[9] De Champlain AF, Margolis MJ, King A, Klass DJ (1997). Standardized patients’ accuracy in recording examinees’ behaviors using checklists. Acad Med.

[10] Bartman I, Smee S, Roy M (2013). A method for identifying extreme OSCE examiners. Clin Teach.

[11] Dainoff MJ, editor. How can we enhance the impact of HFE on the world? Presidential Forum Position Paper. Proceedings of the Human Factors and Ergonomics Society Annual Meeting. SAGE Publications, Thousand Oaks; 2007.

[12] Byrne A, Tweed N, Halligan C (2014). A pilot study of the mental workload of objective structured clinical examination examiners. Med Educ.

[13] Tavares W, Eva K (2013). Exploring the impact of mental workload on rater-based assessments. Adv Health Sci Educ.

[14] Wickens C (2008). Multiple resources and mental workload. Hum Factors.

[15] Byrne A (2011). Measurement of mental workload in clinical medicine: a review study. Anesth Pain Med.

[16] Yurko Y, Scerbo M, Prabhu A, Acker C, Stefanidis D (2010). Higher mental workload is associated with poorer laparoscopic performance as measured by the NASA-TLX tool. Simul Healthc.

[17] Welford A (1978). Mental workload as a function of demand, capacity, strategy and skill.. Ergonomics.

[18] Selby C, Osman L, Davis M, Lee M (1995). How to do it: set up and run an objective structured clinical exam. BMJ.

[19] McKinley DW, Norcini JJ (2014). How to set standards on performance-based examinations: AMEE Guide No. 85. Med Teach.

[20] Noor S, Batra S, Byrne A (2011). Learning opportunities in the clinical setting (LOCS) for medical students: a novel approach. Med Teach.

[21] Ericsson KA. The road to excellence: the acquisition of expert performance in the arts and sciences, sports, and games. Psychology Press, Hove, East Sussex; 2014.

[22] Hope D, Cameron H (2015). Examiners are most lenient at the start of a two-day OSCE. Med Teach.

[23] Vu NV, Marcy MM, Colliver JA, Verhulst SJ, Travis TA, Barrows HS (1992). Standardized (simulated) patients’ accuracy in recording clinical performance check-list items. Med Educ.

[24] van den Eertwegh V, van Dulmen S, van Dalen J, Scherpbier AJJA, van der Vleuten CPM (2013). Learning in context: Identifying gaps in research on the transfer of medical communication skills to the clinical workplace. Patient Educ Couns.

